# Relative inhibitory activities of taniborbactam and xeruborbactam against KPC variants conferring reduced susceptibility or resistance to ceftazidime/avibactam, cefiderocol, and cefepime/taniborbactam

**DOI:** 10.1128/aac.00897-26

**Published:** 2026-09-30

**Authors:** Salud Rodríguez-Pallares, Christophe Le Terrier, Tania Blanco-Martín, Roberto Rilo-Antelo, Cristina Elías-López, Lucía Sánchez-Peña, Carlos Molina-Cáceres, Gloria Pérez-Rodríguez, Lucía González-Pinto, Luis Martínez-Martínez, Laurent Poirel, Germán Bou, Jorge Arca-Suárez

**Affiliations:** 1 Servicio de Microbiología Clínica & Grupo de Investigación en Microbiología, Instituto de Investigación Biomédica de A Coruña (INIBIC), Complexo Hospitalario Universitario de A Coruña (CHUAC), SERGAS. Universidade da Coruña (UDC) 16737, A Coruña, Spain; 2 CIBER de Enfermedades Infecciosas (CIBERINFEC), Instituto de Salud Carlos III 38176, Madrid, Spain; 3 Medical and Molecular Microbiology, Faculty of Science and Medicine, University of Fribourg 27211, Fribourg, Switzerland; 4 Department of Anaesthesiology, Pharmacology, Intensive Care and Emergency Medicine, Faculty of Medicine, University of Geneva 30578 , Geneva, Switzerland; 5 Instituto Maimónides de Investigación Biomédica de Córdoba (IMIBIC) 215147, Córdoba, Spain; 6 Unidad Clínica de Microbiología, Hospital Universitario Reina Sofía 16501, Córdoba, Spain; 7 Departamento de Química Agrícola, Edafología y Microbiología, Universidad de Córdoba 16735, Córdoba, Spain; 8 Swiss National Reference Center for Emerging Antibiotic Resistance (NARA), University of Fribourg 27211, Fribourg, Switzerland; 9 Departamento de Fisioterapia, Medicina y Ciencias Biomédicas, Universidad de A Coruña 16737 , A Coruña, Spain; Universita degli Studi di Roma La Sapienza, Rome, Italy

**Keywords:** ceftazidime-avibactam, β-lactamase, *Klebsiella pneumoniae*, β-lactamase inhibitor, taniborbactam, xeruborbactam, cefiderocol, carbapenemase, KPC

## Abstract

The emergence of KPC variants associated with ceftazidime/avibactam resistance may also compromise the activity of other recently developed β-lactams, including cefiderocol and cefepime/taniborbactam. Xeruborbactam is a boronic acid β-lactamase inhibitor with potent activity against serine- and metallo-β-lactamases and is currently under clinical development in combination with cefiderocol. We evaluated the *in vitro* activity of cefiderocol/xeruborbactam against a panel of clinical *Klebsiella pneumoniae* isolates and isogenic *Escherichia coli* transformants (including both wild-type and porin-deficient backgrounds) producing KPC variants. Xeruborbactam and taniborbactam activities were compared using ceftazidime, cefepime, and cefiderocol as reporter substrates. Inhibitory activity was analyzed by determining the IC₅₀ values for both inhibitors against KPC variants. Most clinical isolates producing KPC variants showed increased MIC values and substantial cross-resistance to ceftazidime/avibactam, cefiderocol, and cefepime/taniborbactam. Cefiderocol/xeruborbactam retained potent activity against all clinical isolates, regardless of the KPC variant produced or the outer membrane permeability status. These findings were confirmed in wild-type and porin-deficient isogenic *E. coli* models, in which the combination of KPC variants and porin loss synergistically increased resistance to multiple β-lactams, while cefiderocol/xeruborbactam consistently restored susceptibility to wild-type levels. Comparative MIC analyses showed that xeruborbactam enhanced the activity of all β-lactam partners to a greater extent than taniborbactam. IC₅₀ determinations revealed a 17-fold to 73-fold greater inhibitory potency of xeruborbactam, which was maintained across structurally diverse KPC variants. Overall, cefiderocol/xeruborbactam exhibited potent activity against KPC-producing Enterobacterales, including isolates producing KPC variants associated with reduced susceptibility or resistance to ceftazidime/avibactam, cefiderocol, and cefepime/taniborbactam.

## INTRODUCTION

Infections caused by KPC-producing Enterobacterales have historically been associated with high mortality rates due to limited therapeutic options (1). This landscape changed with the clinical introduction of ceftazidime/avibactam, the first β-lactam/β-lactamase inhibitor combination found to be active against serine carbapenemase-producing Enterobacterales (2). Previous studies have demonstrated improved patient survival with the use of ceftazidime/avibactam relative to previously available therapies. Accordingly, this combination rapidly became, and remains, a cornerstone for the treatment of infections caused by KPC-producing Enterobacterales (3). However, the widespread use of this combination has led to important collateral effects, including the *in vivo* selection of isolates producing KPC variants conferring resistance to ceftazidime/avibactam due to mutations in the *bla*_KPC_ gene, eventually leading to therapeutic failures (4).

Owing to the structural plasticity of KPC enzymes, more than 290 variants have been described to date. Substitutions within the Ω-loop region are the most frequently identified among those variants involved in ceftazidime/avibactam resistance, although changes in other regions, including the 237-243 loop and 267-275 loop, have also been reported (5). These variants may exert diverse collateral effects, including restoration of susceptibility or increased resistance to other β-lactams (6, 7). Notably, among recently approved or investigational β-lactam agents, cefiderocol and cefepime/taniborbactam often exhibit substantial cross-resistance with ceftazidime/avibactam in clinical *Klebsiella pneumoniae* isolates producing KPC variants. The mechanism underlying cross-resistance to cefiderocol has been characterized for KPC-31 (carrying a D179Y change in the Ω-loop), one of the most frequently selected variants following ceftazidime/avibactam exposure (8). This variant exploits structural similarities between ceftazidime and cefiderocol, which results in reduced *K*_M_ values and promotes the formation of a long-lived covalent intermediate, thereby increasing resistance to both agents (9). A similar mechanism may also contribute to reduced susceptibility to cefepime/taniborbactam through enhanced recognition of cefepime as a substrate. Specifically, the D179Y substitution markedly reduces *K*_M_ values for cefepime, from >500 µM in KPC-2 and KPC-3 to 3.7 and 90 μM, respectively, consistent with the increased cefepime MICs associated with these variants (10). In clinical isolates, these effects may be exacerbated by additional resistance mechanisms, such as decreased outer membrane permeability and efflux pump overproduction, which can act synergistically with KPC alterations to partially compromise cefepime/taniborbactam activity (11).

Xeruborbactam (formerly QPX7728) is a developmental ultra-broad-spectrum boronic acid-derived β-lactamase inhibitor obtained by rational modification of a benzoxaborinine scaffold (12). It exhibits potent inhibitory activity against β-lactamases from all Ambler classes (A, B, C, and D), typically in the nanomolar range, and its spectrum extends beyond that of taniborbactam to include most IMP-type metallo-β-lactamases (13–15). Xeruborbactam also displays modest intrinsic antibacterial activity against Enterobacterales, a feature that may further contribute to its enhanced potentiation of partner β-lactams (16). However, recent studies have also highlighted important limitations in the spectrum of xeruborbactam. Although it inhibits most clinically relevant serine- and metallo-β-lactamases, its activity is reduced against certain IMP variants, including IMP-6 and IMP-10, which harbor the S226G and V67F substitutions, respectively (15). In addition, reduced inhibitory activity has also been reported for a limited number of uncommon metallo-β-lactamases, including SIM-1, SPM-1, PFM-1, and AIM-1 (14). Beyond β-lactamase inhibition, the activity of xeruborbactam is partially compromised in *Pseudomonas aeruginosa* due to active efflux mediated by the MexAB-OprM system, which reduces intracellular xeruborbactam concentrations (17). Xeruborbactam initially entered clinical development in combination with meropenem, and most *in vitro* data have been generated with this combination (18). However, development has shifted toward cefiderocol/xeruborbactam to enhance activity against Gram-negative pathogens and broaden coverage of prevalent resistance mechanisms. This combination is currently under evaluation in phase I clinical trials (ClinicalTrials.gov identifiers: and NCT06547554 and NCT07104162). In previous studies, we demonstrated that xeruborbactam strongly inhibits wild-type KPC β-lactamases produced in both *Escherichia coli* and *P. aeruginosa* backgrounds (17, 19). However, the ability of xeruborbactam to protect cefiderocol against KPC variants associated with enhanced cephalosporin-hydrolyzing activity remains unclear. In this study, we evaluated the *in vitro* activity of cefiderocol in combination with xeruborbactam against clinical *K. pneumoniae* isolates and isogenic *E. coli* strains producing diverse, clinically relevant KPC variants associated with resistance to ceftazidime/avibactam. We also examined the collateral effects on cefiderocol and cefepime/taniborbactam and compared the inhibitory activities of xeruborbactam and taniborbactam against this challenging set of enzymatic targets.

## RESULTS AND DISCUSSION

### Susceptibility of clinical *K. pneumoniae* isolates producing KPC variants to cephalosporins in combination with taniborbactam and xeruborbactam

The MIC values for all agents are summarized in Table 1. The clinical *K. pneumoniae* isolates ARGA00246 and ARGA00247 were included as representatives of epidemic high-risk clones ST307 and ST512, producing KPC-2 and KPC-3, respectively. Isolate ARGA00246 harbored intact OmpK35 and OmpK36 porins, whereas isolate ARGA00247 carried a frameshift mutation in *ompK35*. Both isolates exhibited resistance or decreased susceptibility to ceftazidime, cefepime, and meropenem, while remaining susceptible to ceftazidime/avibactam, cefepime/taniborbactam, cefiderocol, and cefiderocol/xeruborbactam. This susceptibility profile is consistent with the expected activity of these β-lactam and β-lactam/β-lactamase inhibitor combinations against producers of KPC-2 or KPC-3 enzymes (20). Among the combinations tested, cefiderocol/xeruborbactam displayed the highest activity, with MIC values ≤0.03 mg/L for both isolates. This was followed by cefepime/taniborbactam (MICs, 0.25-0.5 mg/L) and ceftazidime/avibactam (MICs, 1-4 mg/L). Notably, cefiderocol/xeruborbactam MICs were 8-fold to 128-fold lower than those of cefepime/taniborbactam and ceftazidime/avibactam, respectively. This enhanced activity of cefiderocol/xeruborbactam likely reflects (i) the high intrinsic stability of cefiderocol to KPC-2- and KPC-3-mediated hydrolysis (21), (ii) the potent inhibitory activity of xeruborbactam against KPC-2 and KPC-3 enzymes, previously found to be in the nanomolar range (22), and (iii) the intrinsic antibacterial activity of xeruborbactam, which targets several PBPs in Enterobacterales (16).

**TABLE 1:** Detailed phenotypic and genotypic features of clinical isolates carrying different KPC variants.

Strain	Bacterial species	ST	KPC β-lactamase	Amino acid alteration with respect to KPC-2 ^*a*^	Location in sequence ^*b*^	MIC (mg/L)^*c*^^,*d*^											Outer membrane porin alterations ^*a*^	
						CAZ (R > 4)	C/A (R > 8)	C/T (R > 4)	C/X (R > 4)	FEP (R > 4)	F/T (R > 4)	F/X (R > 4)	FDC (R > 2)	FDC/T (R > 2)	FDC/X (R > 2)	MEM (R > 8)	OmpK35	OmpK36
ARGA00246	*Klebsiella pneumoniae*	307	KPC-2	NA	NA	128	1	1	0.25	128	0.25	≤0.06	0.125	≤0.03	≤0.03	8	**–**	–
ARGA00247	*Klebsiella pneumoniae*	512	KPC-3	H274Y	NA	1,024	4	2	0.5	64	0.5	0.125	0.25	≤0.03	≤0.03	16	C66fs	–
20220294	*Klebsiella pneumoniae*	512	KPC-66	**L167_∆EL_N170**, H274Y	Ω-loop	1,024	32	32	64	32	2	0.25	2	0.125	0.25	4	C66fs	ins_114_GD
KPLA	*Klebsiella pneumoniae*	258	KPC-35	**L169P**	Ω-loop	1,024	32	32	1	64	2	0.5	0.5	≤0.03	≤0.03	1	C66fs	ins_114_GD
171513	*Klebsiella pneumoniae*	512	KPC-94	**L169H_∆N_S171**, H274Y	Ω-loop	2,048	512	32	16	128	2	0.5	4	0.25	0.125	1	C66fs	ins_114_GD
183358	*Klebsiella pneumoniae*	512	KPC-48	**L169P, A172T**, H274Y	Ω-loop	2,048	128	256	16	128	8	1	32	1	0.5	8	C66fs	ins_114_GD
183111	*Klebsiella pneumoniae*	512	KPC-39	**A172T**, H274Y	Ω-loop	1,024	64	64	2	128	4	0.5	8	1	0.5	16	C66fs	ins_114_GD
202826	*Klebsiella pneumoniae*	512	KPC-85	**A172V**, H274Y	Ω-loop	1,024	32	64	2	128	2	0.25	2	0.5	0.25	64	C66fs	ins_114_GD
180510	*Klebsiella pneumoniae*	512	KPC-95	**A172T, D179Y**, H274Y	Ω-loop	2,048	512	512	512	128	16	1	256	4	1	32	C66fs	ins_114_GD
231595	*Klebsiella pneumoniae*	512	KPC-178	**P174L**, H274Y	Ω-loop	512	32	32	4	128	4	8	1	0.125	≤0.03	32	C66fs	ins_114_GD
20211109	*Klebsiella pneumoniae*	512	KPC-31	**D179Y**, H274Y	Ω-loop	2,048	128	512	2	256	8	0.5	4	2	0.25	2	C66fs	ins_114_GD
966	*Klebsiella pneumoniae*	512	KPC-31	**D179Y**, H274Y	Ω-loop	2,048	256	256	16	128	16	1	4	2	0.5	4	C66fs	ins_114_GD
175871	*Klebsiella pneumoniae*	512	KPC-31	**D179Y**, H274Y	Ω-loop	2,048	512	512	128	128	16	2	256	4	1	4	C66fs	ins_114_GD
183483	*Klebsiella pneumoniae*	512	KPC-31	**D179Y**, H274Y	Ω-loop	2,048	256	256	8	128	8	0.5	4	2	0.125	2	C66fs	ins_114_GD
196226	*Klebsiella pneumoniae*	512	KPC-31	**D179Y**, H274Y	Ω-loop	2,048	128	256	8	128	8	2	8	4	0.5	4	C66fs	ins_114_GD
183860	*Klebsiella pneumoniae*	512	KPC-47	**A172T, T242A**, H274Y	Ω-loop, 237-243 loop	1,024	64	128	2	128	4	0.25	8	1	0.25	32	C66fs	ins_114_GD
HUPM-3	*Klebsiella pneumoniae*	512	KPC-8	**V240G**, H274Y	237-243 loop	2,048	32	32	1	256	4	0.5	2	1	0.25	64	C66fs	ins_114_GD
HUPM-36	*Klebsiella pneumoniae*	512	KPC-8	**V240G**, H274Y	237-243 loop	2,048	16	16	0.5	64	0.5	≤0.06	2	1	0.25	4	C66fs	–
20220166	*Klebsiella pneumoniae*	512	KPC-23	**V240A**, H274Y	237-243 loop	1,024	16	4	0.25	32	0.25	≤0.06	1	0.25	0.125	4	C66fs	–
–231992	*Klebsiella pneumoniae*	512	KPC-28	**Y241_∆GT_A244**, H274Y	237-243 loop	2,048	128	512	2	128	16	0.5	4	0.5	≤0.03	1	C66fs	ins_114_GD
20220366	*Klebsiella pneumoniae*	512	KPC-132	**K273_ ins_ (SRAPNKDDK)_H274**, H274Y	267-275 loop	1,024	64	256	2	256	8	0.5	1	0.5	≤0.03	16	C66fs	ins_114_GD
213842	*Klebsiella pneumoniae*	512	KPC-132	**K273_ ins_ (SRAPNKDDK)_H274**, H274Y	267-275 loop	1,024	64	128	2	256	4	0.5	4	2	0.25	16	C66fs	ins_114_GD
214802	*Klebsiella pneumoniae*	512	KPC-132	**K273_ ins_ (SRAPNKDDK)_H274**, H274Y	267-275 loop	1,024	64	128	0.5	256	4	1	2	0.25	≤0.03	16	C66fs	ins_114_GD
196228	*Klebsiella pneumoniae*	512	KPC-148	**K273_ins_(YSEAVYTRAPNKDDK)_H274**, H274Y	267-275 loop	1,024	128	128	1	128	8	0.5	0.5	0.125	≤0.03	32	C66fs	ins_114_GD

^*a*^ NA, not applicable; ∆: deletion of amino acids; ins, insertion of amino acids; fs: frameshift. Amino acid sequence alterations associated with resistance are indicated in bold. –, no changes detected relative to the wild-type gene sequence. The H274Y substitution is intrinsic to KPC-3 and all its variants.

^*b*^ Location in KPC loops previously indicated by Brunetti F. et al. AAC 2026 (23). NA, not applicable.

^*c*^ EUCAST v.16.1 breakpoints indicated. Clinical breakpoints for combinations that have not yet been approved (ceftazidime/taniborbactam, ceftazidime/xeruborbactam, cefepime/taniborbactam, cefepime/xeruborbactam, cefiderocol/taniborbactam, and cefiderocol/xeruborbactam) were interpreted using those of the β-lactam partner. Avibactam, taniborbactam and xeruborbactam were tested at a fixed concentration of 4 mg/L.

^*d*^ CAZ, ceftazidime; C/A, ceftazidime/avibactam; C/T, ceftazidime/taniborbactam; C/X, ceftazidime/xeruborbactam; FEP, cefepime; F/T, cefepime/taniborbactam; F/X, cefepime/xeruborbactam; FDC, cefiderocol; FDC/T, cefiderocol/taniborbactam; FDC/X, cefiderocol/xeruborbactam; MEM, meropenem.

Analysis of MIC reductions using ceftazidime and cefepime as reporter substrates against these strains showed that taniborbactam reduced MIC values with a potency comparable to that of avibactam. When taniborbactam was combined with ceftazidime, MICs decreased from 128-1,024 mg/L to 1-2 mg/L, respectively, while cefepime MICs were reduced from 128-64 mg/L to 0.25-0.5 mg/L. By contrast, xeruborbactam demonstrated higher inhibitory potency, reducing ceftazidime MICs to 0.25 and 0.5 mg/L, and cefepime MICs to ≤0.06 and 0.125 mg/L. Overall, xeruborbactam produced an additional 4-fold to 8-fold reduction in MIC values relative to taniborbactam and avibactam, suggesting an enhanced inhibitory potency *in vitro* against KPC-2 and KPC-3 enzymes.

Clinical *K. pneumoniae* isolates producing KPC variants associated with ceftazidime/avibactam resistance exhibited high-level resistance to ceftazidime (MICs, 512-2,048 mg/L). While cefepime MICs were also generally elevated, meropenem MICs were more variable, consistent with the previously reported collateral effects of KPC variants on carbapenem susceptibility. These variants were detected in clinical isolates, in which we not only observed increased ceftazidime/avibactam MICs (16-512 mg/L) but also reduced activity of cefepime/taniborbactam (MICs, 0.25-16 mg/L) and cefiderocol (MICs, 0.5-256 mg/L), supporting substantial cross-resistance in these recently developed agents. By contrast, cefiderocol/xeruborbactam retained potent activity against all clinical isolates tested, with MIC values ranging from ≤0.03 to 1 mg/L. This finding is particularly notable, given the complex resistance background of these isolates. Consistent with their assignment to the KPC-3-producing ST258/ST512 lineage, which reached the Iberian Peninsula in 2012 and was subsequently disseminated nationwide, the isolates produced diverse KPC variants and exhibited markedly reduced outer membrane permeability, a phenomenon commonly reported in this clone (24, 25). Specifically, all isolates carried inactivating alterations in OmpK35, and all but three harbored the characteristic GD insertion in loop 3 of OmpK36, a modification associated with porin channel narrowing and impaired β-lactam uptake (26).

Comparative analysis using ceftazidime, cefepime, and cefiderocol as reporter substrates revealed marked differences in the inhibitory potency of taniborbactam and xeruborbactam. When combined with ceftazidime, taniborbactam reduced MIC values from 512-2,048 mg/L to 4-512 mg/L (4-fold to 256-fold reductions), whereas xeruborbactam decreased the MICs to 0.25-512 mg/L, achieving reductions ranging from 4-fold to 4,096-fold. This observation is particularly notable, given that these variants are frequently optimized for ceftazidime recognition and trapping, exhibiting very low ceftazidime *K*_M_ values and thereby limiting the capacity of β-lactamase inhibitors to block its binding (27). A similar pattern was observed with cefepime, with taniborbactam reducing MICs from 32-256 mg/L to 0.25-16 mg/L (8-fold to 128-fold), and xeruborbactam consistently decreasing MIC values to ≤0.06-8 mg/L, achieving reductions ranging from 16-fold to ≥1,024-fold. Finally, taniborbactam reduced cefiderocol MICs from 0.5-256 mg/L to ≤0.03-4 mg/L, whereas xeruborbactam consistently reduced MICs to ≤0.03-1 mg/L across all isolates. Altogether, these results indicate that xeruborbactam provides substantially greater potentiation of cephalosporin activity, particularly for cefiderocol, restoring activity against genetically diverse KPC variants even in the presence of major permeability defects.

### Susceptibility of wild-type and porin-deficient isogenic *E. coli* isolates producing KPC variants to ceftazidime, cefepime, and cefiderocol combined with taniborbactam or xeruborbactam

To further assess the direct contribution of KPC variants to the resistance phenotypes observed in clinical isolates, the corresponding enzymes were produced in two isogenic *E. coli* backgrounds (Table 2): *E. coli* TG1, with wild-type outer membrane permeability, and *E. coli* HB4, lacking the major porins OmpF and OmpC. Consistent with the findings in clinical isolates, transformants producing wild-type KPC-2 and KPC-3 remained susceptible to cefiderocol, and cefepime/taniborbactam in both genetic backgrounds. By contrast, in most cases, production of KPC variants resulted in increased MICs of ceftazidime, ceftazidime/avibactam, cefiderocol, and cefepime/taniborbactam, confirming the direct contribution of these enzymes to cross-resistance against these agents. As expected, this effect was markedly amplified in the *E. coli* HB4 background, supporting the cooperative contribution of KPC-mediated resistance and reduced permeability. In this strain, the ceftazidime MICs were much higher than for *E. coli* TG1, increasing from 8-2,048 mg/L to 64->2,048 mg/L, depending on the variant produced. A similar shift was observed for ceftazidime/avibactam, with MICs increasing from 0.5-128 mg/L in *E. coli* TG1 to 2-1,024 mg/L in *E. coli* HB4. Cefepime MICs also increased markedly, from 1-64 mg/L in *E. coli* TG1 to 8-1,024 mg/L in *E. coli* HB4, while meropenem MICs increased from ≤0.06-8 mg/L to ≤0.06-64 mg/L.

**TABLE 2:** Antibiotic susceptibility data of β-lactams and β-lactam/β-lactamase inhibitor combinations against isogenic *E. coli* TG1 and HB4 transformants producing KPC variants

KPC β-lactamase	Amino acid alteration respect to KPC-2 ^*a*^	Location in sequence ^*b*^	MIC (mg/L) *^c^**^,^^d^*																					
			*E. coli* TG1											*E. coli* HB4										
			CAZ (R > 4)	C/A (R > 8)	C/T (R > 4)	C/X (R > 4)	FEP (R > 4)	F/T (R > 4)	F/X (R > 4)	FDC (R > 2)	FDC/T (R > 2)	FDC/X (R > 2)	MEM (R > 8)	CAZ (R > 4)	C/A (R > 8)	C/T (R > 4)	C/X (R > 4)	FEP (R > 4)	F/T (R > 4)	F/X (R > 4)	FDC (R > 2)	FDC/T (R > 2)	FDC/X (R > 2)	MEM (R > 8)
*E. coli* TG1	NA	NA	0.25	≤0.06	≤0.06	≤0.06	≤0.06	≤0.06	≤0.06	≤0.03	≤0.03	≤0.03	≤0.06	–	–	–	–	–	–	–	–	–	–	–
*E. coli* HB4	NA	NA	–	–	–	–	–	–	–	–	–	–	–	1	0.25	0.5	0.25	0.5	0.5	0.25	≤0.03	≤0.03	≤0.03	0.125
KPC-2	NA	NA	64	0.5	0.25	≤0.06	16	≤0.06	≤0.06	0.125	≤0.03	≤0.03	4	512	4	8	0.5	512	2	0.5	0.25	≤0.03	≤0.03	64
KPC-3	H274Y	NA	128	0.5	0.25	≤0.06	16	≤0.06	≤0.06	0.125	≤0.03	≤0.03	2	2,048	16	16	0.5	1,024	2	0.5	0.5	≤0.03	≤0.03	64
KPC-66	**L167_∆EL_N170**, H274Y	Ω-loop	256	8	2	0.125	2	≤0.06	≤0.06	0.5	≤0.03	≤0.03	≤0.06	2,048	64	64	1	32	2	0.25	1	≤0.03	≤0.03	1
KPC-35	**L169P**	Ω-loop	128	4	2	≤0.06	2	≤0.06	≤0.06	0.5	≤0.03	≤0.03	0.125	1,024	16	8	0.5	64	1	0.25	1	≤0.03	≤0.03	1
KPC-94	**L169H_∆N_S171**, H274Y	Ω-loop	1,024	128	4	0.5	16	≤0.06	≤0.06	2	≤0.03	≤0.03	≤0.06	>2,048	1,024	64	8	256	2	0.5	4	≤0.03	≤0.03	0.5
KPC-48	**L169P, A172T**, H274Y	Ω-loop	256	64	8	0.25	16	0.25	≤0.06	0.5	≤0.03	≤0.03	0.125	2,048	128	64	1	64	4	0.25	1	0.06	≤0.03	1
KPC-39	**A172T**, H274Y	Ω-loop	32	8	0.5	≤0.06	16	≤0.06	≤0.06	0.06	≤0.03	≤0.03	0.25	256	16	8	0.5	64	1	0.25	0.25	≤0.03	≤0.03	4
KPC-85	**A172V**, H274Y	Ω-loop	16	1	0.125	≤0.06	1	≤0.06	≤0.06	≤0.03	≤0.03	≤0.03	0.125	64	2	2	0.25	8	0.5	0.125	0.06	≤0.03	≤0.03	1
KPC-95	**A172T, D179Y**, H274Y	Ω-loop	256	32	2	≤0.06	2	≤0.06	≤0.06	1	≤0.03	≤0.03	≤0.06	2,048	64	64	0.5	64	2	0.125	2	0.06	≤0.03	0.5
KPC-178	**P174L**, H274Y	Ω-loop	128	8	2	≤0.06	16	≤0.06	≤0.06	0.125	≤0.03	≤0.03	0.5	256	16	8	0.5	64	0.5	0.125	0.25	≤0.03	≤0.03	8
KPC-31	**D179Y**, H274Y	Ω-loop	512	32	8	0.125	8	≤0.06	≤0.06	2	≤0.03	≤0.03	0.125	2,048	256	64	1	128	4	0.5	2	0.125	≤0.03	1
KPC-47	**A172T, T242A**, H274Y	Ω-loop, 237-243 loop	256	16	8	≤0.06	16	≤0.06	≤0.06	0.5	≤0.03	≤0.03	0.5	>2,048	256	128	1	256	4	0.25	2	0.125	≤0.03	32
KPC-8	**V240G**, H274Y	237-243 loop	2,048	8	8	≤0.06	64	≤0.06	≤0.06	0.5	≤0.03	≤0.03	4	>2,048	128	64	1	1,024	4	0.5	2	0.125	≤0.03	64
KPC-23	**V240A**, H274Y	237-243 loop	2,048	2	2	≤0.06	64	≤0.06	≤0.06	0.5	≤0.03	≤0.03	8	>2,048	32	32	0.5	1,024	4	0.25	1	≤0.03	≤0.03	64
KPC-28	**Y241_∆GT_A244**, H274Y	237-243 loop	512	32	16	≤0.06	16	≤0.06	≤0.06	2	≤0.03	≤0.03	0.125	>2,048	256	256	1	256	8	1	4	0.5	≤0.03	1
KPC-132	**K273_ ins_ (SRAPNKDDK)_H274**, H274Y	267-275 loop	256	16	4	0.125	16	≤0.06	≤0.06	0.25	≤0.03	≤0.03	0.125	2,048	256	64	0.5	256	4	0.125	1	0.06	≤0.03	8
KPC-148	**K273_ins_(YSEAVYTRAPNKDDK)_H274**, H274Y	267-275 loop	128	32	4	≤0.06	16	≤0.06	≤0.06	0.25	≤0.03	≤0.03	0.5	1,024	256	64	0.5	256	8	0.125	1	0.125	≤0.03	32

^*a*^ NA, not applicable; ∆, deletion of amino acids; ins: insertion of amino acids. Amino acid sequence alterations associated with resistance are indicated in bold. The H274Y substitution is intrinsic to KPC-3 and all its variants.

^*b*^ Location in KPC loops previously indicated by Brunetti F. et al. AAC 2026 (23). NA: not applicable.

^*c*^ EUCAST v.16.1 breakpoints indicated. Clinical breakpoints for combinations that have not yet been approved (ceftazidime/taniborbactam, ceftazidime/xeruborbactam, cefepime/taniborbactam, cefepime/xeruborbactam, cefiderocol/taniborbactam, and cefiderocol/xeruborbactam) were interpreted using those of the β-lactam partner. Avibactam, taniborbactam and xeruborbactam were tested at a fixed concentration of 4 mg/L. –, MIC not determined.

^*d*^ CAZ, ceftazidime; C/A, ceftazidime/avibactam; C/T, ceftazidime/taniborbactam; C/X, ceftazidime/xeruborbactam; FEP, cefepime; F/T, cefepime/taniborbactam; F/X, cefepime/xeruborbactam; FDC, cefiderocol; FDC/T, cefiderocol/taniborbactam; FDC/X, cefiderocol/xeruborbactam; MEM, meropenem.

Notably, the activity of cefepime/taniborbactam was significantly compromised in the low-permeability *E. coli* HB4 background. While MIC values in *E. coli* TG1 were generally low (≤0.06-0.25 mg/L), they increased to 0.5-8 mg/L in *E. coli* HB4 across multiple KPC variants, in some cases approaching or exceeding the proposed susceptibility breakpoints. These findings indicate that taniborbactam may not fully overcome the high level of cefepime MICs due to the combined effects of KPC variant production and reduced permeability. Similar effects of the interplay between β-lactamase production and reduced permeability on cefepime/taniborbactam resistance have been observed in clinical *K. pneumoniae* strains producing NDM-type carbapenemases (28). Cefiderocol MICs were affected in *E. coli* TG1 (≤0.03-2 mg/L) and increased only modestly in *E. coli* HB4 (0.06-4 mg/L). The MIC increase was more limited than observed for other β-lactams, suggesting that impaired outer membrane permeability has a limited impact on this siderophore cephalosporin. This observation is consistent with the siderophore-mediated uptake mechanism, which may partly bypass permeability defects (29). Consistent with the findings in clinical isolates, cefiderocol/xeruborbactam retained the highest activity among all combinations tested, with very low MICs obtained (≤0.03 mg/L in *E. coli* TG1 and in *E. coli* HB4 transformants) across the entire panel, irrespective of the KPC variant produced.

Comparative analysis using ceftazidime, cefepime, and cefiderocol as reporter substrates further highlighted the greater potentiating effect of xeruborbactam. With ceftazidime, taniborbactam reduced MICs in *E. coli* TG1 transformants from 8-2,048 mg/L to 0.125-16 mg/L, whereas xeruborbactam decreased them to ≤0.06-0.5 mg/L. A similar trend was observed in the *E. coli* HB4 transformants, which exhibit higher baseline MICs due to the interplay between β-lactamase production and impaired outer membrane permeability. In these strains, taniborbactam reduced ceftazidime MICs from 64->2,048 mg/L to 2-256 mg/L, while xeruborbactam lowered them to 0.25-8 mg/L. This finding is particularly relevant because many KPC variants are optimized for ceftazidime binding and trapping, as exemplified by the classic D179Y substitution, present in natural variants such as KPC-31, which exhibits a marked reduction in *K*_M_ values (from >2,000 µM to <5 µM) relative to the wild-type enzyme (30).

A similar pattern was observed with cefepime. In *E. coli* TG1 transformants, both taniborbactam and xeruborbactam restored cefepime activity, leading to very low MIC values (generally ≤0.06 mg/L). However, in the *E. coli* HB4 background, cefepime/taniborbactam MICs ranged from 0.5-8 mg/L and were significantly affected by reduced outer membrane permeability, whereas cefepime/xeruborbactam MICs remained lower (≤0.06-1 mg/L). Thus, xeruborbactam provided more consistent restoration of cefepime activity under reduced permeability conditions. When cefiderocol was used as a reporter, taniborbactam was found to reduce MICs, particularly in the *E. coli* TG1 background, with values reduced from ≤0.03-2 mg/L to ≤0.03 mg/L in most transformants. In the *E. coli* HB4 background, MICs were reduced from 0.125-4 mg/L to ≤0.03-0.5 mg/L. By contrast, cefiderocol/xeruborbactam consistently restored MICs to ≤0.03 mg/L in both *E. coli* TG1 and *E. coli* HB4 transformants. This corresponded to reductions of up to 64-fold in *E. coli* TG1 and up to 128-fold in *E. coli* HB4 for variants with elevated baseline MICs. This confirms that xeruborbactam potentiates its β-lactam partner more effectively than taniborbactam against KPC variants, regardless of the permeability background.

### Relative inhibitory activities of taniborbactam and xeruborbactam against KPC β-lactamases

Determination of IC₅₀ values against KPC β-lactamases revealed a consistently greater inhibitory potency of xeruborbactam than that of taniborbactam across the panel tested (Table 3). Xeruborbactam exhibited markedly lower IC₅₀ values than taniborbactam against wild-type KPC-2 and KPC-3 enzymes (0.03-0.05 µM vs 1.16-1.25 µM), corresponding to a 23-fold to 42-fold increase in inhibitory potency. A similar pattern was observed with the KPC variants, with xeruborbactam maintaining low IC₅₀ values in the nanomolar range (0.02-0.06 µM) and taniborbactam IC₅₀ values remaining close to the micromolar range (0.65-2.93 µM). This translated into consistent differences in inhibitory activity, with xeruborbactam showing 17-fold to 73-fold lower IC₅₀ values depending on the variant. Notably, amino acid substitutions in the KPC enzymes tested had a limited impact on the inhibitory activity of either compound, as IC₅₀ values remained within comparable ranges across wild-type and variant enzymes. Overall, these findings indicate that xeruborbactam provides more robust and consistent inhibition across structurally diverse KPC variants than taniborbactam. This biochemical profile aligns with the enhanced restoration of β-lactam activity observed in susceptibility assays, particularly for cefiderocol/xeruborbactam.

**TABLE 3:** Determination of the 50% inhibitory concentration (IC_50_) of xeruborbactam according to different subclasses and variants of KPC enzymes, in comparison to taniborbactam^*d*^

KPC β-lactamase ^*a*^	Amino acid alteration respect to KPC–2 ^*b*^	Location in sequence ^*c*^	IC_50_ taniborbactam (µM)	IC_50_ xeruborbactam (µM)	Relative IC_50_ (Taniborbactam/Xeruborbactam)
KPC-2	NA	NA	1.16	0.05	23.20
KPC-3	H274Y	NA	1.25	0.03	41.67
KPC-66	**L167_∆EL_N170**, H274Y	Ω-loop	–	–	–
KPC-35	**L169P**	Ω-loop	–	–	–
KPC-94	**L169H_∆N_S171**, H274Y	Ω-loop	–	–	–
KPC-48	**L169P, A172T**, H274Y	Ω-loop	–	–	–
KPC-39	**A172T**, H274Y	Ω-loop	1.35	0.03	45.00
KPC-85*	**A172V**, H274Y	Ω-loop	0.65	0.03	21.67
KPC-95	**A172T, D179Y**, H274Y	Ω-loop	–	–	–
KPC-178	**P174L**, H274Y	Ω-loop	1.41	0.03	47.00
KPC-31	**D179Y**, H274Y	Ω-loop	–	–	–
KPC-47	**A172T, T242A**, H274Y	Ω-loop, 237-243 loop	2.93	0.04	73.25
KPC-8	**V240G**, H274Y	237-243 loop	1.06	0.05	21.20
KPC-23	**V240A**, H274Y	237-243 loop	1.02	0.03	34.00
KPC-28	**Y241_∆GT_A244**, H274Y	237-243 loop	0.87	0.02	43.50
KPC-132	**K273_ ins_ (SRAPNKDDK)_H274**, H274Y	267-275 loop	1.18	0.05	23.60
KPC-148	**K273_ins_(YSEAVYTRAPNKDDK)_H274**, H274Y	267-275 loop	1.03	0.06	17.17

^*a*^ *For those enzymes, nitrocefin was used as a substrate, considering that cephalothin was not hydrolyzed by those enzymes.

^*b*^ NA, not applicable; ∆, deletion of amino acids; ins: insertion of amino acids. Amino acid sequence alterations associated with resistance are indicated in bold. The H274Y substitution is intrinsic to KPC-3 and all its variants.

^*c*^ Location in KPC loops previously indicated by Brunetti F. et al*. *AAC 2026 (23). NA, not applicable.

^*d*^ –, no measurable substrate hydrolysis was observed, precluding the determination of IC₅₀ values.

Notably, amino acid substitutions in KPC enzymes associated with resistance to ceftazidime/avibactam evaluated here had limited impact on the IC_50_ values of either taniborbactam or xeruborbactam. This observation may be explained by the selective pressure exerted by ceftazidime/avibactam, in which ceftazidime acts as the primary β-lactam selecting agent. Accordingly, many KPC variants appear to evolve toward conferring enhanced ceftazidime resistance rather than toward reduced susceptibility to the accompanying inhibitor, namely, avibactam (31). These observations have been corroborated by combined crystallographic analysis and molecular dynamics simulation studies, in which the Ω-loop, the 237-243 loop, and the 267-275 loop conformations have been found to be associated with increased ceftazidime “trapping” (32). Thus, although KPC variants may show collateral decreased avibactam susceptibility, the primary mechanism of ceftazidime/avibactam resistance in these variants relies on improved cephalosporin binding (33). Here, we demonstrate the limited impact of KPC substitutions associated with ceftazidime/avibactam resistance on the inhibitory activity of taniborbactam and xeruborbactam. These findings indicate that the structural determinants governing inhibition by cyclic boronates may be preserved despite alterations within the Ω-loop, the 237-243 loop, and the 267-275 loop of KPC enzymes. Taniborbactam and xeruborbactam primarily act as transition-state analogs that mimic the tetrahedral high-energy intermediate generated during β-lactam hydrolysis. Structural analyses have shown that these inhibitors form reversible tetrahedral boronate adducts with the catalytic S70, occupying the oxyanion hole and reproducing the geometry of the catalytic transition state (34). This distinctive mode of inhibition may explain why many KPC variants associated with ceftazidime/avibactam retain high susceptibility to cyclic boronates. In addition, recent structural studies of xeruborbactam with class A β-lactamases have demonstrated highly stable S70-boronate complexes with slow deborylation kinetics and extensive hydrophobic stabilization, features that may contribute to the increased potency of xeruborbactam against these enzymes (35). Future comparative structural and kinetic analyses of taniborbactam and xeruborbactam with KPC variants would provide valuable insights into the superior activity of xeruborbactam. Collectively, these findings reinforce the potential of cyclic boronate β-lactamase inhibitors, particularly xeruborbactam, as robust partners for β-lactams against KPC-producing pathogens. An important question remains whether KPC variants conferring resistance to cyclic boronates will emerge following their clinical introduction. Based on our findings, resistance to cefepime/taniborbactam is likely to arise preferentially through permeability defects, whereas resistance to cefiderocol/xeruborbactam may additionally involve alterations in iron uptake pathways, as previously described.

Overall, our findings demonstrate the consistent activity of cefiderocol/xeruborbactam against both isogenic and clinical strains producing diverse KPC variants associated with resistance or reduced susceptibility to ceftazidime/avibactam, cefepime/taniborbactam, and cefiderocol. This activity likely results from the intrinsic high internalization in the bacterial cell through iron-uptake pathways, together with the intrinsic stability of cefiderocol to β-lactamase-mediated hydrolysis, and the potent inhibitory activity of xeruborbactam, which consistently outperformed taniborbactam against the different KPC enzymes. These results support the continued clinical development of cefiderocol/xeruborbactam as a promising therapeutic option against multidrug-resistant KPC-producing Enterobacterales, including those harboring resistance-associated variants selected during ceftazidime/avibactam therapy.

## MATERIALS AND METHODS

### Clinical isolates

We analyzed a collection of 24 KPC-producing *K. pneumoniae* isolates encompassing a broad diversity of KPC variants. The panel included the wild-type enzymes KPC-2 and KPC-3, as well as 15 additional variants harboring amino acid substitutions, insertions, or deletions associated with ceftazidime/avibactam resistance (KPC-8, KPC-23, KPC-28, KPC-31, KPC-35, KPC-39, KPC-47, KPC-48, KPC-66, KPC-85, KPC-94, KPC-95, KPC-132, KPC-148, and KPC-178). The alterations in these variants mapped to key structural regions implicated in ceftazidime/avibactam resistance, including the Ω-loop, 237-243 loop, and 267-275 loop. The isolates were previously recovered from patients exposed to ceftazidime/avibactam in high KPC-endemic settings or identified in the context of nationwide surveillance studies of carbapenemase-producing Enterobacterales (CPE) conducted in Spain.

### Whole-genome sequencing

Total genomic DNA from clinical isolates was obtained using a Wizard Genomic DNA Purification Kit (Promega). Quality control of paired-end Illumina reads was performed using fastp (v0.32.2) and BMTagger (v3.1) (36, 37). Genome assemblies were generated using Unicycler (v0.5.0) (38). Assembly quality, completeness, and contamination were assessed using CheckM (v1.1.3) (39). Species identification was conducted using KmerFinder (v3.2) and multilocus sequence typing (MLST; Center for Genomic Epidemiology, v2.0). Putative open reading frames were annotated using Bakta (v1.7.0) (40, 41). Acquired antimicrobial resistance genes were identified using Resistance Gene Identifier (RGI; v5.2.0) and the Comprehensive Antibiotic Resistance Database (CARD; v3.2.8) (42). Chromosomal mutations in genes encoding penicillin-binding proteins (PBPs), porins, efflux pump regulators, and iron uptake systems were analyzed by variant calling using Snippy (v4.6.0), with *K. pneumoniae* ATCC 10031 (β-lactam-susceptible) and a closely related KPC-31-producing ST512 *K. pneumoniae* isolate (20211109) as reference genomes (43). Naturally occurring missense variants in canonical chromosomal genes previously associated with β-lactam resistance (e.g., *mrdA*, *ftsI*, *ramA*, *marA*, *acrR*, and *tolC*) were manually reviewed and excluded from further analysis.

### Construction of recombinant strains

We engineered a collection of wild-type (*E. coli* TG1) and porin-deficient (*E. coli* HB4) *E. coli* strains producing KPC-2, KPC-3, and the corresponding KPC variants found in the above-described clinical isolates section to precisely assess the impact of KPC variants identified in clinical isolates on β-lactam resistance, as well as to evaluate their interplay with reduced outer membrane permeability on the activity of β-lactams and β-lactam/β-lactamase inhibitor combinations. To this end, genes encoding the different KPC-like enzymes were amplified by PCR, cloned into the pUCP24 plasmid, and expressed in both *E. coli* hosts, as previously described (18). The resulting transformants were validated by Sanger sequencing of recombinant plasmids, plasmid restriction analysis, and antimicrobial susceptibility testing.

### Antimicrobial susceptibility testing

The MICs of ceftazidime, ceftazidime/avibactam, ceftazidime/taniborbactam, ceftazidime/xeruborbactam, cefepime, cefepime/taniborbactam, cefepime/xeruborbactam, cefiderocol, cefiderocol/taniborbactam, cefiderocol/xeruborbactam, and meropenem were determined in triplicate for all clinical and recombinant isolates. MIC testing was conducted with cation-adjusted Mueller-Hinton (MH) broth for all agents except cefiderocol, cefiderocol/taniborbactam, and cefiderocol/xeruborbactam, which were tested in iron-depleted cation-adjusted MH broth, prepared according to CLSI M100 guidelines, using BD-BBL brand MH broth. For β-lactam/β-lactamase inhibitor combinations, avibactam, taniborbactam, and xeruborbactam were tested at a fixed concentration of 4 mg/L. EUCAST v.16.0 clinical breakpoints and guidelines (http://www.eucast.org/clinical_breakpoints/) were used for reference purposes. For unapproved combinations, the clinical breakpoint of the β-lactam partner alone was applied. Reference strains *E. coli* ATCC 25922, *K. pneumoniae* ATCC 700603, *E. coli* NCTC 13353, and *P. aeruginosa* ATCC 27853 were used as controls. The potency of xeruborbactam was validated in all assays by testing it in combination with meropenem against *K. pneumoniae* strain ATCC BAA-2814 (meropenem/xeruborbactam MIC range= 0.015-0.06 mg/L), as recommended by the CLSI 2025 guidelines.

### Determination of IC₅₀ values for taniborbactam and xeruborbactam against KPC β-lactamases

Recombinant *E. coli* TG1 transformants harboring recombinant plasmids encoding different KPC β-lactamases were inoculated into 50 mL of Luria-Bertani broth supplemented with 30 mg/L gentamicin and grown overnight at 37°C. Bacterial cells were harvested by centrifugation, resuspended in 10 mL of 100 mM phosphate buffer (pH 7.0), and lysed by sonication using a Vibra-Cell 75186 sonicator (Thermo Fisher Scientific). The lysate was then centrifuged for 1 h at 11,000 × *g* at 4°C. The presence of β-lactamase activity was confirmed using nitrocefin (200 µM). Kinetic measurements were performed at room temperature in 100 mM sodium phosphate buffer (pH 7.0) using a UV-visible ULTROSPEC 2100 Pro spectrophotometer (Amersham Biosciences, Buckinghamshire, UK). IC₅₀ values for taniborbactam and xeruborbactam were determined in triplicate. Different concentrations of each inhibitor were preincubated with crude enzyme extracts for 3 min at room temperature to determine the concentration required to reduce the hydrolysis rate of 100 µM cephalothin by 50%. Cephalothin hydrolysis was monitored at 262 nm using a molar extinction coefficient (Δε) of -7,960 M⁻¹·cm⁻¹. To ensure the comparability of β-lactamase inhibitory activity, lysate concentrations were adjusted to yield similar cephalothin hydrolysis rates, defined as those producing slopes between 0.06 and 0.1 OD/min, as previously described (15).
